# Supplementary material for: Lot quality assurance sampling survey for water, sanitation and hygiene monitoring and evidence-based advocacy in Bentiu IDP camp, South Sudan
Source: PLoS One. 2024 Jul 15;19(7):e0302712. doi: 10.1371/journal.pone.0302712 (PMC11249214; doi:10.1371/journal.pone.0302712)
Supplement: S1 File — (DOCX) [file pone.0302712.s001.docx]

## Comparison to the previous LQAS Surveys in Bentiu IDP (Monitoring and evaluation)

The WatSan LQAS survey has been implemented in Bentiu PoC (current IDP) in 2014, 2017 and then in 2019. Hence, to compare the results from those LQAS surveys would help to see either an improvement or deterioration of the WASH situation in the camp. The following table compares the results with the previous three surveys. We have added the confidence intervals for the current year and the most recent previous survey for the ease of comparison.

### Water supply and coverage indicators

Table 12. Comparison of the current findings of the water supply and coverage indicators to the previous LQAS Surveys in Bentiu IDP camp, August 2021.

| Indicator | | 2014 | 2017 | 2019 | | 2021 | | Target | |
| --- | --- | --- | --- | --- | --- | --- | --- | --- | --- |
| Proportion of households that report using a portable water source for drinking in dry AND rainy season | | 95.4% | 100.0% | 93.3% (88.1-98.5) | | 95%  (90.5-99.5) | | 95% | |
| Proportion of households that report using PUR or AQUATAB sachets to treat rainwater | | 72.5% | 16.6% | N/A | | N/A | | 95% | |
| Proportion of households that report that water was available from their water source at least six of the seven days | | 70.1% | 50.4% | 72.6% (64.5-80.6) | | 68.90% (60.8-77.1) | | 95% | |
| Proportion of households that report that they always get their containers filled from the tap stand before the water will stop running | | N/A | N/A | 61.8% (53.7-69.9) | | 51.4% (44.1-77.1) | | 95% | |
| Proportion of household that find the taste of the water from the tap stand acceptable | | 15.9% | 49.6% | 42.0% (32.1-51.9) | | 65.5% (55.6-75.3) | | 75% | |
| Proportion of households that report using a potable water source for cooking | | 93.6% | 99.1% | 100.0% (NA) | | 99.3% (NA) | | 95% | |
| Proportion of households that report using a potable water source for washing dishes | | 86.9% | 97.9% | 100.0%  (NA) | | 100% (NA) | | 95% | |
| Proportion of households that report using a potable water source for washing your hands | | 90.7% | 97.8% | 99.0% (96.9-100) | | 100% (NA) | | 95% | |
| Proportion of households that report using a potable water source for washing your clothes | | 54.2% | 55.8% | 71.8% (63.1-80.5) | | 69.8% (60.6-79.0) | | 65% | |
| Proportion of households that report using a potable water source for bathing | | 64.8% | 95.5% | 100.0% (NA) | | 94.40% (89.3-99.6) | | 80% | |
| Proportion of households that have at least one water container that can hold water | | N/A | 82.5% | 94.8% (90.4-99.3) | | 95.50% (91.7-99.4) | | 95% | |
| Proportion of households that had at least 40L of water the day before | | N/A | N/A | 85.8% (78.5-93.3) | | 81.90% (75.7-88.1) | | 95% | |
| Proportion of households that keep water in containers for less than one day | | 15.9% | 33.2% | 39.6% (29.5-49.8) | | 36.60% (26.4-46.8) | | 95% | |
| **Legend** |  | | | |  | |  | |  |
|  | Significant deterioration from the latest LQAS | | | |  | |  | |  |
|  | Stable compared to the latest LQAS (95% CI overlaps) | | | | | | | |  |
|  | Not applicable (100% positive achievement) | | | | | | | |  |

### Hygiene coverage indicators

Table 13. Comparison of the current finding of sanitation coverage indicators to the previous LQAS surveys in Bentiu IDP camp, August 2021.

| Indicator | 2014 | 2017 | 2019 | 2021 | Target |
| --- | --- | --- | --- | --- | --- |
| Proportion of households that report having their own water jug for cleansing after defecation | 78.6% | 31.2% | 58.6% (41.1-76) | 66.80%  (49-84.6%) | 95% |
| Proportion of households that have a hand washing area within their living area | 49.3% | 11.5% | 16.4% (8.9-23.9%) | 17.90% (10.9-24.8%) | 95% |
| Proportion of households that can show at least one piece of soap | 78.3% | 26.9% | 44.8% (35-54.5%) | 26.40% (17.4%-35.3%) | 95% |
| Proportion of households that have been visited by a hygiene promoter within the last week | 66.1% | 97.3% | 83.1% (75.7-90.5%) | 49% (39.6-58.5%) | 95% |
| Proportion of households that do NOT eat from a shared plate | 6.7% | 3.9% | 4.2% (0.5-7.9%) | 6.5% (1.5-11.6%) | 75% |
| Proportion of households that do NOT wash a dead body AND do NOT wash hands in a shared bowl at a funeral | 17.5% | 21.9% | 54.0% (44.6-63.4%) | 57.6 % (48.4-66.7%) | 95% |

| **Legend** |  |  |  |
| --- | --- | --- | --- |
|  | Significant deterioration from the latest LQAS difference) |  |  |
|  | Stable compared to the latest LQAS (95% CI overlaps) | | |
|  | Not applicable (100% positive achievement) | | |

### Sanitation related indicators

Table 14. Comparison of the current finding of sanitation coverage indicators to the previous LQAS surveys in Bentiu IDP camp, August 2021.

| **Indicator** | **2014** | **2017** | **2019** | **2021** | **Target** |
| --- | --- | --- | --- | --- | --- |
| Proportion of households that report using an improved sanitation facility | 82.3% | 100.0% | 100% (NA) | 90.50% (84.7-96.4%) | 95% |
| Proportion of households whose sanitation facility is observed to be in an acceptable condition | 6.9% | 3.7% | 24.8%  (17.4-32.2%) | 22.80% (15.6-30.1% | 90% |
| Proportion of households that have an acceptable hand washing area by the toilet facility they use | 32.7% | 11.7% | 11.7%  (5-18.4%) | 13.20% (6.6-19.9%) | 90% |
| Proportion of households whose female members use acceptable materials for menstrual hygiene | N/A | N/A | 82.8%  (76.2%-89.3%) | 97.40% (94.5-100%) | 95% |

| **Legend** |  |  |  |
| --- | --- | --- | --- |
|  | Deterioration from the latest LQAS ( there is significant difference) |  |  |
|  | Stable compared to the latest LQAS (95% CI overlaps) | | |
|  | Not applicable (100% positive achievement) | | |

### Health related indicators

Table 15. Comparison of the current findings prevalence of WASH related diseases to the previous LQAS Surveys in Bentiu IDP camp, August 2021.

| Indicator | 2014 | 2017 | 2019 | 2021 | Minimum Target coverage |
| --- | --- | --- | --- | --- | --- |
| Prevalence of NO diarrhoea in children <5 years in last two weeks | 38.7% | 40.7% | 61.6%  (51.6-71.6%) | 59.7% (49.6-69.7%) | 90% |
| Prevalence of NO eye infection in children <5 years in last two weeks | 35.4% | 44.3% | 83.1%  (75.6-90.6%) | 71.3% (62.1-80.6%) | 90% |
| Prevalence of NO ear infection in children <5 years in last two weeks | 51.0% | 54.8% | 94.0%  (89.2-98.8%) | 91.2% (85.1-97.2%) | 90% |
| Prevalence of NO skin infection in children <5 years in last two weeks | 57.9% | 49.9% | 95.7%  (93.8%-97.6%) | 89.5% (83.9-95.2%) | 90% |

| **Legend** |  |  |  |
| --- | --- | --- | --- |
|  | Deterioration from the latest LQAS ( there is significant difference) |  |  |
|  | Stable compared to the latest LQAS (95% CI overlaps) | | |
|  | Not applicable (100% positive achievement) | | |
