# Supplementary material for: Lot quality assurance sampling survey for water, sanitation and hygiene monitoring and evidence-based advocacy in Bentiu IDP camp, South Sudan
Source: PLoS One. 2024 Jul 15;19(7):e0302712. doi: 10.1371/journal.pone.0302712 (PMC11249214; doi:10.1371/journal.pone.0302712)
Supplement: S2 File — (DOCX) [file pone.0302712.s002.docx]

## Appendix III- Target Indicators

**Table 1 – Water indicators**

| **Number** | **Indicator** | **Definition** | **Questionnaire Answer** | **Target %** |
| --- | --- | --- | --- | --- |
| **Water 1** | Proportion of households that report using a potable water source for drinking both in dry and rainy season | Tap stand used throughout the year | W1 Answer 1 AND W2 Answer 1 | 95 |
| **Water 2** | Proportion of households that report using PUR or AQUATAB sachets to treat rainwater | Households that use rainwater as drinking water and treat it with chlorination sachets | W3 Answer 1 | 95 |
| **Water 3** | Proportion of households that report that water was available from their water source at least six of the seven days | No outage for ≥1 day | W5 Answers 1 AND 2 | 95 |
| **Water 4** | Proportion of households that report that they always get their containers filled from the tap stand before the water will stop running | All containers filled | W7 Answer 1 | 95 |
| **Water 5** | Proportion of household that find the taste of the water from the tap stand acceptable | Like the taste | W9 Answer 1 | 75 |
| **Water 6** | Proportion of households that report using a potable water source for cooking | Tap stand | W10 Answer 1 | 95 |
| **Water 7** | Proportion of households that report using a potable water source for washing dishes | Tap stand | W11 Answer 1 | 95 |
| **Water 8** | Proportion of households that report using a potable water source for washing your hands | Tap stand | W12 Answer 1 | 95 |
| **Water 9** | Proportion of households that report using a potable water source for washing your clothes | Tap stand | W13 Answer 1 | 65 |
| **Water 10** | Proportion of households that report using a potable water source for bathing | Tap stand | W14 Answer 1 | 80 |
| **Water 11** | Proportion of households that have at least one water container that can hold water | At least one container observed, not broken (able to hold water) | W18 value ≥ 1 | 95 |
| **Water 12** | Proportion of households that had at least 40L of water the day before | Observe the containers, which are not broken, estimate their volume and ask how many times they were filled the day before. The total volume of the previous day should be at least 40L | For each observed container multiply the volume (W20) by the number of times filled the day before (W21). Then add to find the total volume. | 95 |
| **Water 13** | Proportion of households that keep water in containers for less than one day | Store water in household for less than one day | W22 Answer 1 | 95 |

**Table 2 – Hygiene Indicators**

| **Number** | **Indicator** | **Definition** | **Questionnaire Answer** | **Target %** |
| --- | --- | --- | --- | --- |
| **Hygiene 1** | Proportion of households that report having their own water jug for cleansing after defecation | Use of water for cleansing after defecation | H1 Answer 1 | 90 |
| **Hygiene 2** | Proportion of households that have a hand washing area within their living area | Water and soap present, within the confines of their living area | H3 Answer 1 AND  H4 Answer 1 | 70 |
| **Hygiene 3** | Proportion of households that can show at least one piece of soap | At least one piece of soap seen by interviewer | H9 Answers 2-4 | 95 |
| **Hygiene 4** | Proportion of households that have been visited by a hygiene promoter within the last week | Visited by hygiene promoter within the last week | H10 Answer 1 | 95 |
| **Hygiene 5** | Proportion of households that do NOT eat from a shared plate |  | H12 Answer 2 | 65 |
| **Hygiene 6** | Proportion of households that do NOT wash a dead body AND do NOT wash hands in a shared bowl at a funeral |  | H13 Answer 2  AND  H14 Answer 2 | 95 |

**Table 3 – Sanitation Indicators**

| **Number** | **Indicator** | **Definition** | **Questionnaire Answer** | **Target %** |
| --- | --- | --- | --- | --- |
| **Sanitation 1** | Proportion of households that report using an improved sanitation facility | Simple pit latrine | SA1 Answer 1 | 95 |
| **Sanitation 2** | Proportion of households whose sanitation facility is observed to be in an acceptable condition | It has a door, it seems safe, has no visible faeces and it is not full | SA4 Answer 1 AND Answer 2 AND Answer 3 AND Answer 4 | 90 |
| **Sanitation 3** | Proportion of households that have an acceptable hand washing area by the toilet facility they use | Water and soap present | SA7 Answer 1 AND  SA8 Answer 1 | 90 |
| **Sanitation 4** | Proportion of households whose female members use acceptable materials for menstrual hygiene | Disposable or reusable cloth/pad used for menstrual hygiene | SA9 Answers 1-3 OR other appropriate method mentioned in OTHER | 95 |

**Table 4 – Waterborne disease indicators**

| **Number** | **Indicator** | **Definition** | **Questionnaire Answer** | **Target %** |
| --- | --- | --- | --- | --- |
| **Disease 1** | Prevalence of diarrhoea among children <5 years in the last two weeks | Diarrhoea **NOT** reported | D3 Answer 2 | 90 |
| **Disease 2** | Prevalence of eye infection among children <5 years in the last two weeks | Eye infection **NOT** reported | D4 Answer 2 | 90 |
| **Disease 3** | Prevalence of ear infection among children <5 years in the last two weeks | Ear infection **NOT** reported | D5 Answer 2 | 90 |
| **Disease 4** | Prevalence of skin infection among children <5 years in the last two weeks | Skin infection **NOT** reported | D6 Answer 2 | 90 |
