# Supplementary material for: Lot quality assurance sampling survey for water, sanitation and hygiene monitoring and evidence-based advocacy in Bentiu IDP camp, South Sudan
Source: PLoS One. 2024 Jul 15;19(7):e0302712. doi: 10.1371/journal.pone.0302712 (PMC11249214; doi:10.1371/journal.pone.0302712)
Supplement: S3 File — (DOCX) [file pone.0302712.s003.docx]

# Annex IV. Questionnaire

# Identifiers:

| **No.** | **HOUSEHOLD IDENTIFICATION** | **Answers** |
| --- | --- | --- |
| 1 | Sector and block | S____B____ |
| 2 | Household ID (1 – 19) | ______ |
| 3 | Team number | ______ |
| 4 | Date of interview | ______ /______ /______ |
| Demographics: |  |  |
| **No.** | **DEMOGRAPHICS** | **Answers** |
| 1 | How many people slept here last night? | ______ |
| 2 | How many children less than 5 years old live here | ______ |
| 3 | How many people more than 50 years old live here? | ______ |
| 4 | Female head of household  **SELECT ONLY ONE RESPONSE** | YES  NO |
| 5 | Sex of respondent  **SELECT ONLY ONE RESPONSE** | FEMALE  MALE |

**Questionnaire 1 for Households**

**Section 1: Water supply and storage**

| **No.** | **Questions** | **Answers** | **Coding** | **Skips** |
| --- | --- | --- | --- | --- |
| W1 | What is the **main** source of **drinking** water for members of this household during the **dry** season?  **SELECT ONLY ONE RESPONSE** | TAPSTAND  POND  STAGNANT WATER  OTHER (SPECIFY)  __________ | 1  2  3  4 |  |
| W2 | What is the **main** source of **drinking** water for members of this household during the **rainy** season?  **SELECT ONLY ONE RESPONSE** | TAPSTAND  POND  RAINWATER  STAGNANT WATER  OTHER (SPECIFY)  __________ | 1  2  3  4  5 |  |
| **If W2 is 3 (RAINWATER), then ask W3. If both W1 and W2 are NOT 1 (TAPSTAND) then skip to W10** | | | | |
| W3 | Do you ever use PUR or AQUATAB sachets to treat rainwater? | YES  NO | 1  2 |  |
| W4 | Which tap stand? (Your water source is located in which Sector and Block?)  **SELECT ALL RESPONSES MENTIONED**  **COMPLETE THE BLOCK NUMBER** | S1 Block ___  S2 Block___  S3 Block___  S4 Block___  S5 Block ___  HOSPITAL COMPOUND  BASE LOG OR UNMISS COMPOUND  OTHER (SPECIFY)  __________ | 1  2  3  4  5  6  7  8 |  |
| W5 | In the last week, how often was water available from this source?  **SELECT ONLY ONE RESPONSE** | EVERYDAY  SIX DAYS  LESS THAN SIX DAYS | 1  2  3 |  |
| W6 | How many times per day the tap stand you use has water running? | ONE  TWO  MORE THAN TWO TIMES | 1  2  3 |  |
| W7 | Do you always get all your containers filled from the tap stand before the water will stop running? | YES  NO | 1  2 | **🡪 W9** |
| W8 | If NO, then what do you do MOST OF THE TIMES?  **SELECT ONLY ONE RESPONSE** | WAIT UNTIL THE NEXT SERVICE  USE ANOTHER TAPSTAND (OTHER BLOCK SECTOR/ HOSPITAL/UNMISS)  COLLECT WATER FROM ANY OTHER SOURCE | 1  2  3 |  |
| W9 | Do you find the taste of the water from the tap stand acceptable | YES  NO | 1  2 |  |
| W10 | What is the **main** source of water which you use for **cooking** for the members of this household?  **SELECT ONLY ONE RESPONSE** | TAPSTAND  POND  RAINWATER  STAGNANT WATER  OTHER (SPECIFY)  __________ | 1  2  3  4  5 |  |
| W11 | What is the **main** source of water which you use for **washing dishes** for the members of this household?  **SELECT ONLY ONE RESPONSE** | TAPSTAND  POND  RAINWATER  STAGNANT WATER  OTHER (SPECIFY)  __________ | 1  2  3  4  5 |  |
| W12 | What is the **main** source of water which you use for **washing your hands** for members of this household?  **SELECT ONLY ONE RESPONSE** | TAPSTAND  POND  RAINWATER  STAGNANT WATER  OTHER (SPECIFY)  __________ | 1  2  3  4  5 |  |
| W13 | What is the **main** source of water which you use for **washing your clothes** for members of this household?  **SELECT ONLY ONE RESPONSE** | TAPSTAND  POND  RAINWATER  STAGNANT WATER  OTHER (SPECIFY)  __________ | 1  2  3  4  5 |  |
| W14 | What is the **main** source of water which you use for **bathing** for members of this household?  **SELECT ONLY ONE RESPONSE** | TAPSTAND  POND  RAINWATER  STAGNANT WATER  OTHER (SPECIFY)  __________ | 1  2  3  4  5 |  |
| W15 | Do you have any water containers that are used in this household? | YES  NO | 1  2 | **🡪 H1 if NO** |
| W16 | May we see these water containers? | YES  NO | 1  2 | **🡪 H1 if NO** |
| W17 | OBSERVE: How many water containers are there?  **SELECT ONLY ONE RESPONSE** | ZERO CONTAINERS  ONE CONTAINER  TWO CONTAINERS  THREE CONTAINERS  FOUR CONTAINERS OR MORE | 1  2  3  4  5 | **🡪 H1 if NO** |
| W18 | OBSERVE: How many containers can hold water (not broken)?  **WRITE THE NUMBER OF CONTAINERS** | NOT BROKEN (ABLE TO HOLD WATER) | _ |  |
| W19 | OBSERVE: How is the appearance of each container (not broken)?  **SELECT ALL RESPONSES THAT APPLY FOR EACH CONTAINER**  **(Build into Kobo to correspond to total containers in W18)** | HAS A NARROW MOUTH (<10CM)  HAS A LID/ SECURE FITTING COVER  HAS A TAP  CLEAN (NO VISIBLE DIRT)  NONE OF THE ABOVE | 1  2  3  4  5 |  |
| W20 | OBSERVE: What is the capacity of each container (not broken)?  **WRITE THE VOLUME OF EACH CONTAINER**  **(Build into Kobo to correspond to total containers in W18)** | APPROXIMATE VOLUME (LITRES) | _ |  |
| W21 | How many times each container (not broken) was filled with water yesterday?  **WRITE THE NUMBER OF TIMES FILLED FOR EACH CONTAINER**  **(Build into Kobo to correspond to total containers in W18)** | TIMES FILLED YESTERDAY | _ |  |
| W22 | For how many days do you keep the water in the containers?  **SELECT ONLY ONE RESPONSE** | LESS THAN A DAY  1 DAY  2 DAYS  3 DAYS  MORE THAN 3 DAYS | 1  2  3  4  5 |  |

**Section 2: Hygiene**

| H1 | Do you have a water jug for cleansing after defecation which only used by your household? | YES  NO | 1  2 |  |
| --- | --- | --- | --- | --- |
| H2 | May we see where members of your household most often wash their hands?  **SELECT ONLY ONE RESPONSE** | YES  NO- PERMISSION NOT GIVEN  NOT POSSIBLE TO OBSERVE | 1  2  3 | **🡪 H5**  **🡪 H5** |
| H3 | OBSERVE: Is there is soap or detergent available where they wash their hands? | YES  NO | 1  2 |  |
| H4 | OBSERVE: Is there water available where they wash their hands? | YES  NO | 1  2 |  |
| H5 | Do you have soap in this household? | YES  NO | 1  2 | **🡪 H10** |
| H6 | Where do you get your soap from? | SOAP DISTRIBUTION  MARKET  HOSPITAL/CLINIC  HEALTH PROMOTER  OTHER (SPECIFY)  __________ | 1  2  3  4  5 |  |
| H7 | How many pieces of soap do you have?  **SELECT ONLY ONE RESPONSE** | ONE PIECE  TWO PIECES  THREE PIECES OR MORE | 1  2  3 |  |
| H8 | May we see your soap? | YES  NO | 1  2 | **🡪 H10** |
| H9 | OBSERVE: How many pieces of soap are there? (Do not forget to count any soap piece observed in the handwashing area)  **SELECT ONLY ONE RESPONSE** | ZERO PIECE  ONE PIECE  TWO PIECES  THREE PIECES OR MORE | 1  2  3  4 |  |
| H10 | Have you ever been visited by a hygiene promoter within the last week? | YES  NO | 1  2 |  |
| H11 | Do you wash your hands in a shared bowl before eating? | YES  NO | 1  2 |  |
| H12 | Do you eat food from a shared plate? | YES  NO | 1  2 |  |
| H13 | Do you wash the dead body at a funeral? | YES  NO | 1  2 |  |
| H14 | Do you wash your hands in a shared bowl at a funeral? | YES  NO | 1  2 |  |

**Section 3: Sanitation**

| SA1 | Where do you go for the toilet? | PIT LATRINE  BESIDE THE LATRINE  BUSH  NEAR THE POND  ANY OPEN SPACE  NEAR THE HOUSE  OTHER (SPECIFY)  ____________________ | 1  2  3  4  5  6  7 | **🡪 SA3** |
| --- | --- | --- | --- | --- |
| SA2 | Why don’t you use the latrine?  **SELECT ALL THE RESPONSES THAT APPLY** | I AM NOT FAMILIAR  IT IS DIRTY  IT IS TOO HOT INSIDE  AGAINST MY CULTURE/BELIEFS  OTHER (SPECIFY)  ____________________ | 1  2  3  4  5 |  |
| SA3 | May I see the pit latrine? | YES  NO | 1  2 | **🡪 SA9** |
| SA4 | OBSERVE: Is there a latrine? | YES  NO | 1  2 | **🡪 SA9** |
| SA5 | OBSERVE: How is the latrine?  **SELECT ALL THE RESPONSES THAT APPLY** | DOOR IN PLACE AND IN USE  SLAB AND PIT WALLS ENSURING  SAFETY FOR THE USERS  NO VISIBLE FAECES AROUND  NOT FULL | 1  2  3  4 |  |
| SA6 | OBSERVE: Is there a handwashing area? | YES  NO | 1  2 |  |
| SA7 | OBSERVE: Is there water available in the handwashing area? | YES  NO | 1  2 |  |
| SA8 | OBSERVE: Is soap or detergent available in the handwashing area? | YES  NO | 1  2 |  |
| SA9 | What do you use for menstrual hygiene?  N.B. Ensure you get a female interviewee from the household for this question  **SELECT ALL RESPONSES THAT APPLY** | REUSABLE CLOTH  DISPOSABLE CLOTH  PAD  OTHER (SPECIFY) _____________ | 1  2  3  4 |  |

**Questionnaire 2: for Parents/Guardians of a child under 5 years**

**Section 4: WASH Related Diseases for CHILDREN UNDER 5 YEARS**

| D1 | In the last two weeks, has this child been sick? | YES  NO | 1  2 | **🡪 END** |
| --- | --- | --- | --- | --- |
| D2 | What symptoms has this child had in the last two weeks? | 1__________________  2__________________  3__________________  4__________________  5__________________  **USE THE SYMPTOMS REPORTED TO COMPLETE D3-D5, DO NOT ASK D3-D5** |  |  |
| D3 | **MARK (DO NOT ASK):**  Was diarrhoea reported in this child? | YES  NO | 1  2 |  |
| D4 | **MARK (DO NOT ASK):**  Was eye infection reported in this child? | YES  NO | 1  2 |  |
| D5 | **MARK (DO NOT ASK):**  Was ear infection reported in this child? | YES  NO | 1  2 |  |
| D6 | **MARK (DO NOT ASK):**  Was skin infection reported in this child? | YES  NO | 1  2 |  |
| **END** | | | | |

## 11.3 Appendix 4- Indicators

**WASH Indicators - Definitions**

**Bentiu IDP, May 2021**

**Table 1 – Water indicators**

| **Number** | **Indicator** | **Definition** | **Questionnaire Answer** | **Target %** |
| --- | --- | --- | --- | --- |
| **Water 1** | Proportion of households that report using a potable water source for drinking both in dry and rainy season | Tap stand used throughout the year | W1 Answer 1 AND W2 Answer 1 | 95 |
| **Water 2** | Proportion of households that report using PUR or AQUATAB sachets to treat rainwater | Households that use rainwater as drinking water and treat it with chlorination sachets | W3 Answer 1 | 95 |
| **Water 3** | Proportion of households that report that water was available from their water source at least six of the seven days | No outage for ≥1 day | W5 Answers 1 AND 2 | 95 |
| **Water 4** | Proportion of households that report that they always get their containers filled from the tap stand before the water will stop running | All containers filled | W7 Answer 1 | 95 |
| **Water 5** | Proportion of household that find the taste of the water from the tap stand acceptable | Like the taste | W9 Answer 1 | 75 |
| **Water 6** | Proportion of households that report using a potable water source for cooking | Tap stand | W10 Answer 1 | 95 |
| **Water 7** | Proportion of households that report using a potable water source for washing dishes | Tap stand | W11 Answer 1 | 95 |
| **Water 8** | Proportion of households that report using a potable water source for washing your hands | Tap stand | W12 Answer 1 | 95 |
| **Water 9** | Proportion of households that report using a potable water source for washing your clothes | Tap stand | W13 Answer 1 | 65 |
| **Water 10** | Proportion of households that report using a potable water source for bathing | Tap stand | W14 Answer 1 | 80 |
| **Water 11** | Proportion of households that have at least one water container that can hold water | At least one container observed, not broken (able to hold water) | W18 value ≥ 1 | 95 |
| **Water 12** | Proportion of households that had at least 40L of water the day before | Observe the containers which are not broken, estimate their volume and ask how many times they were filled the day before. The total volume of the previous day should be at least 40L | For each observed container multiply the volume (W20) by the number of times filled the day before (W21). Then add to find the total volume. | 95 |
| **Water 13** | Proportion of households that keep water in containers for less than one day | Store water in household for less than one day | W22 Answer 1 | 95 |

**Table 2 – Hygiene Indicators**

| **Number** | **Indicator** | **Definition** | **Questionnaire Answer** | **Target %** |
| --- | --- | --- | --- | --- |
| **Hygiene 1** | Proportion of households that report having their own water jug for cleansing after defecation | Use of water for cleansing after defecation | H1 Answer 1 | 90 |
| **Hygiene 2** | Proportion of households that have a hand washing area within their living area | Water and soap present, within the confines of their living area | H3 Answer 1 AND  H4 Answer 1 | 70 |
| **Hygiene 3** | Proportion of households that can show at least one piece of soap | At least one piece of soap seen by interviewer | H9 Answers 2-4 | 95 |
| **Hygiene 4** | Proportion of households that have been visited by a hygiene promoter within the last week | Visited by hygiene promoter within the last week | H10 Answer 1 | 95 |
| **Hygiene 5** | Proportion of households that do NOT eat from a shared plate |  | H12 Answer 2 | 65 |
| **Hygiene 6** | Proportion of households that do NOT wash a dead body AND do NOT wash hands in a shared bowl at a funeral |  | H13 Answer 2  AND  H14 Answer 2 | 95 |

**Table 3 – Sanitation Indicators**

| **Number** | **Indicator** | **Definition** | **Questionnaire Answer** | **Target %** |
| --- | --- | --- | --- | --- |
| **Sanitation 1** | Proportion of households that report using an improved sanitation facility | Simple pit latrine | SA1 Answer 1 | 95 |
| **Sanitation 2** | Proportion of households whose sanitation facility is observed to be in an acceptable condition | It has a door, it seems safe, has no visible faeces and it is not full | SA4 Answer 1 AND Answer 2 AND Answer 3 AND Answer 4 | 90 |
| **Sanitation 3** | Proportion of households that have an acceptable hand washing area by the toilet facility they use | Water and soap present | SA7 Answer 1 AND  SA8 Answer 1 | 90 |
| **Sanitation 4** | Proportion of households whose female members use acceptable materials for menstrual hygiene | Disposable or reusable cloth/pad used for menstrual hygiene | SA9 Answers 1-3 OR other appropriate method mentioned in OTHER | 95 |

**Table 4 – Waterborne disease indicators**

| **Number** | **Indicator** | **Definition** | **Questionnaire Answer** | **Target %** |
| --- | --- | --- | --- | --- |
| **Disease 1** | Prevalence of diarrhoea among children <5 years in the last two weeks | Diarrhoea **NOT** reported | D3 Answer 2 | 90 |
| **Disease 2** | Prevalence of eye infection among children <5 years in the last two weeks | Eye infection **NOT** reported | D4 Answer 2 | 90 |
| **Disease 3** | Prevalence of ear infection among children <5 years in the last two weeks | Ear infection **NOT** reported | D5 Answer 2 | 90 |
| **Disease 4** | Prevalence of skin infection among children <5 years in the last two weeks | Skin infection **NOT** reported | D6 Answer 2 | 90 |
