# Supplementary material for: Lot quality assurance sampling survey for water, sanitation and hygiene monitoring and evidence-based advocacy in Bentiu IDP camp, South Sudan
Source: PLoS One. 2024 Jul 15;19(7):e0302712. doi: 10.1371/journal.pone.0302712 (PMC11249214; doi:10.1371/journal.pone.0302712)
Supplement: S4 File — (DOCX) [file pone.0302712.s004.docx]

## WASH related indicators by sector

### Water supply coverage indicators

Table 7. Water supply indicators by sector in Bentiu IDP camp, August 2021.

| **Indicator** | **S1** | **S2** | **S3** | **S4** | **S5** |  | **Weighted average** | **Weighted (DR)** | **Tar**  **get** | **Target (DR)** |
| --- | --- | --- | --- | --- | --- | --- | --- | --- | --- | --- |
| Proportion of households that report using a potable water source for drinking both in dry and rainy season | 17 | 19 | 18 | 18 | 18 |  | **95.0%** | **16** | **95%** | **16** |
| Proportion of households that report using PUR or AQUATAB sachets to treat rainwater | 0 | 0 | 0 | 0 | 0 |  | **N/A** | **–** | **95%** | **16** |
| Proportion of households that report that water was available from their water source at least six of the seven days | 12 | 14 | 15 | 4 | 17 |  | **68.9%** | **11** | **95%** | **16** |
| Proportion of households that report that they always get their containers filled from the tap stand before the water will stop running | 15 | 16 | 7 | 11 | 5 |  | **51.4%** | **8** | **95%** | **16** |
| Proportion of household that find the taste of the water from the tap stand acceptable | 15 | 11 | 11 | 15 | 12 |  | **65.5%** | **10** | **75%** | **12** |
| Proportion of households that report using a potable water source for cooking | 18 | 19 | 19 | 19 | 19 |  | **99.3%** | **N/A** | **95%** | **16** |
| Proportion of households that report using a potable water source for washing dishes | 19 | 19 | 19 | 19 | 19 |  | **100.0%** | **N/A** | **95%** | **16** |
| Proportion of households that report using a potable water source for washing your hands | 19 | 19 | 19 | 19 | 19 |  | **100.0%** | **N/A** | **95%** | **16** |
| Proportion of households that report using a potable water source for washing their clothes | 19 | 17 | 13 | 12 | 9 |  | **69.8%** | **11** | **65%** | **10** |
| Proportion of households that report using a potable water source for bathing | 19 | 19 | 17 | 19 | 17 |  | **94.4%** | **N/A** | **80%** | **13** |
| Proportion of households that have at least one water container that can hold water | 17 | 19 | 19 | 17 | 18 |  | **95.5%** | **16** | **95%** | **16** |
| Proportion of households that had at least 40L of water the day before | 4 | 19 | 18 | 19 | 14 |  | **81.9%** | **14** | **95%** | **16** |
| Proportion of households that keep water in containers for less than one day | 3 | 12 | 6 | 5 | 6 |  | **36.6%** | **5** | **95%** | **16** |

### Hygiene coverage indicators by sector

Table 8. Hygiene practice and coverage indicators by sector in Bentiu IDP camp, August 2021

| **Indicator** | **S1** | **S2** | **S3** | **S4** | **S5** | **Weighted average** | **Average (DR)** | **Target coverage** | **Target (DR)** |
| --- | --- | --- | --- | --- | --- | --- | --- | --- | --- |
| Proportion of households that report having their own water jug for cleansing after defecation | 15 | 15 | 14 | 9 | 11 | 66.8% | 11 | 95% | 15 |
| Proportion of households that have a hand washing area within their living area | 10 | 6 | 3 | 0 | 1 | 17.9% | 1 | 95% | 11 |
| Proportion of households that can show at least one piece of soap | 6 | 4 | 3 | 4 | 8 | 26.4% | 3 | 95% | 16 |
| Proportion of households that have been visited by a hygiene promoter within the last week | 6 | 10 | 15 | 7 | 6 | 49.0% | 7 | 95% | 16 |
| Proportion of households that do **NOT** eat from a shared plate† | 1 | 1 | 0 | 1 | 3 | 6.5% | N/A | 75% | 10 |
| Proportion of households that do **NOT** wash a dead body AND do NOT wash hands in a shared bowl at a funeral | 16 | 13 | 4 | 13 | 13 | 57.6% | 9 | 95% | 16 |

### Sanitation indicator coverage by sector

Table 9. Coverage of sanitation indicators by sector in Bentiu IDP camp, August 2019.

| **Indicator** | **S 1** | **S2** | **S3** | **S 4** | **S5** | **Weighted average** | **Average (DR)** | **Target coverage** | **Target (DR)** |
| --- | --- | --- | --- | --- | --- | --- | --- | --- | --- |
| Proportion of households that report using an improved sanitation facility | 16 | 19 | 15 | 17 | 19 | 90.5% | 16 | 95% | 16 |
| Proportion of households whose sanitation facility is observed to be in an acceptable condition | 1 | 3 | 2 | 14 | 3 | 22.8% | 2 | 90% | 15 |
| Proportion of households that have an acceptable hand washing area by the toilet facility they use† | 0 | 5 | 6 | 0 | 0 | 13.2% | N/A | 90% | 15 |
| Proportion of households whose female members use acceptable materials for menstrual hygiene | 18 | 16 | 19 | 19 | 19 | 97.4% | N/A | 95% | 16 |

### Health indicators by sector

Table 10. Prevalence of WASH related disease indicators by sector in Bentiu IDP camp, August 2021

| **Indicator** | **S1** | **S2** | **S3** | **S4** | **S5** | **Weighted average** | **Average (DR)** | **Target coverage** | **Target (DR)** |
| --- | --- | --- | --- | --- | --- | --- | --- | --- | --- |
| Proportion of parents/guardians who report NOT having diarrhoea among children <5 years in last two weeks | 12 | 13 | 13 | 10 | 9 | 59.7% | 9 | 90% | 15 |
| Proportion of parents/guardians who report NOT having eye infection among children <5 years in last two weeks | 16 | 13 | 14 | 17 | 10 | 71.3% | 12 | 90% | 15 |
| Proportion of parents/guardians who report NOT having ear infection among children <5 years in last two weeks | 19 | 19 | 15 | 17 | 18 | 91.2% | 16 | 90% | 15 |
| Proportion of parents/guardians who report NOT having skin infection among children <5 years in last two weeks | 18 | 19 | 14 | 16 | 19 | 89.5% | 15 | 90% | 15 |

## Priorities for each sector

Table 11. Priority indicators for poor WASH condition by sector in Bentiu IDP camp, August 2021.

| **Indicator** | **Overall** | **Sector 1** | **Sector 2** | **Sector 3** | **Sector 4** | **Sector 5** |
| --- | --- | --- | --- | --- | --- | --- |
| **Water indicators** | | | | | | |
| Proportion of households that report using a potable water source for drinking in dry AND rainy season | N | - | - | - | - | - |
| Proportion of households that report using PUR or AQUATAB sachets to treat rainwater | N/A | N/A | N/A | N/A | N/A | N/A |
| Proportion of households that report that water was available from their water source at least six of the seven days | Y | 2 | 2 | 2 | 1 | - |
| Proportion of households that report that they always get their containers filled from the tap stand before the water will stop running | Y | - | - | 1 |  | 1 |
| Proportion of household that find the taste of the water from the tap stand acceptable | Y | 2 | 2 | 2 | 1 | 1 |
| Proportion of households that report using a potable water source for cooking | N | - | - | - | - | - |
| Proportion of households that report using a potable water source for washing dishes | N | - | - | - | - | - |
| Proportion of households that report using a potable water source for washing their hands | N | - | - | - | - | - |
| Proportion of households that report using a potable water source for washing their clothes | N | - | - | - |  | 1 |
| Proportion of households that report using a potable water source for bathing | N | - | - | - | - | - |
| Proportion of households that have at least one water container that can hold water | N | - | - | - | - | - |
| Proportion of households that had at least 40L of water the day before | Y | 1 | - |  | - |  |
| Proportion of households that keep water in containers for less than one day | Y | 1 | 2 | 2 | 2 | 1 |
| **Hygiene Indicators** | | | | | | |
| Proportion of households that report having their own water jug for cleansing after defecation | Y | - | - | 2 | 1 | 2 |
| Proportion of households that have a hand washing area within their living area | Y | 2 | 1 | 1 | 1 | 1 |
| Proportion of households that can show at least one piece of soap | Y | 1 | 1 | 1 | 1 | 1 |
| Proportion of households that have been visited by a hygiene promoter within the last week | Y | 1 | 1 | 2 | 1 | 1 |
| Proportion of households that do NOT eat from a shared plate | Y | 1 | 1 | 1 | 1 | 1 |
| Proportion of households that do NOT wash a dead body AND do NOT wash hands in a shared bowl at a funeral | Y | - | 2 | 1 | 2 | 2 |
| **Sanitation Indicators** | | | | | | |
| Proportion of households that report using an improved sanitation facility | Y | - | - | 2 | - | - |
| Proportion of households whose sanitation facility is observed to be in an acceptable condition | Y | 1 | 2 | 2 | -- | 2 |
| Proportion of households that have an acceptable hand washing area by the toilet facility they use | Y | 1 | 1 | 1 | 1 | 1 |
| Proportion of households whose female members use acceptable materials for menstrual hygiene | N | - | - | - | - | - |
| **Disease indicators** | | | | | | |
| Prevalence of NO diarrhoea in children <5 years in last two weeks | Y | 2 | 2 | 2 | 2 | 2 |
| Prevalence of NO eye infection in children <5 years in last two weeks | Y | 2 | 1 | - | - | - |
| Prevalence of NO ear infection in children <5 years in last two weeks | N | - | - | - | - | - |
| Prevalence of NO skin infection in children <5 years in last two weeks | y | - | - | 2 | - | - |
| Y = Attention required;  N = No attention required;  1 = Primary priority;  2 = Secondary priority | | | | | | |
