# Supplementary material for: Lot quality assurance sampling survey for water, sanitation and hygiene monitoring and evidence-based advocacy in Bentiu IDP camp, South Sudan
Source: PLoS One. 2024 Jul 15;19(7):e0302712. doi: 10.1371/journal.pone.0302712 (PMC11249214; doi:10.1371/journal.pone.0302712)
Supplement: Supplementary file 5 [file pone.0302712.s005.docx]

LQAS Table: Decision Rules for Sample Sizes of 12-30 and Coverage Targets/Average of 10%-95%

Sample Size*	**Average Coverage (Baselines) / Annual Coverage Target (Monitoring and Evaluation)**																		
	10%	15%	20%	25%	30%	35%	40%	45%	50%	55%	60%	65%	70%	75%	80%	85%	90%	95%	
12	N/A	N/A	1	1	2	2	3	4	5	5	6	7	7	8	8	9	10	11	
13	N/A	N/A	1	1	2	3	3	4	5	6	6	7	8	8	9	10	11	11	
14	N/A	N/A	1	1	2	3	4	4	5	6	7	8	8	9	10	11	11	12	
15	N/A	N/A	1	2	2	3	4	5	6	6	7	8	9	10	10	11	12	13	
16	N/A	N/A	1	2	2	3	4	5	6	7	8	9	9	10	11	12	13	14	
17	N/A	N/A	1	2	2	3	4	5	6	7	8	9	10	11	12	13	14	15	
18	N/A	N/A	1	2	2	3	5	6	7	8	9	10	11	11	12	13	14	16	
19	N/A	N/A	1	2	3	4	5	6	7	8	9	10	11	12	13	14	15	16	
20	N/A	N/A		2	3	4	5	6	7	8	9	11	12	13	14	15	16		17
21	N/A	N/A	1	2	3	4	5	6	8	9	10	11	12	13	14	16	17		18
22	N/A	N/A	1	2	3	4	5	7	8	9	10	12	13	14	15	16	18		19
23	N/A	N/A	1	2	3	4	6	7	8	10	11	12	13	14	16	17	18		20
24	N/A	N/A	1	2	3	4	6	7	9	10	11	13	14	15	16	18	19		21
25	N/A	1	2	2	4	5	6	8	9	10	12	13	14	16	17	18	20		21
26	N/A	1	2	3	4	5	6	8	9	11	12	14	15	16	18	19	21		22
27	N/A	1	2	3	4	5	7	8	10	11	13	14	15	17	18	20	21		23
28	N/A	1	2	3	4	5	7	8	10	12	13	15	16	18	19	21	22		24
29	N/A	1	2	3	4	5	7	9	10	12	13	15	17	18	20	21	23		25
30	N/A	1	2	3	4	5	7	9	11	12	14	16	17	19	20	22	24		26

N/A: *Not Applicable*, meaning LQAS can not be used in this assessment because the coverage is either too low or too high to assess an SA. This table assumes the lower threshold is 30 percentage points below the upper threshold.

(yellow) : light-shaded cells indicate where *alpha* or *beta* errors are greater than or equal to 10%.

(grey) : dark-shaded cells indicate where *alpha* or *beta* errors are greater than15%.
