# Supplementary material for: Lot quality assurance sampling survey for water, sanitation and hygiene monitoring and evidence-based advocacy in Bentiu IDP camp, South Sudan
Source: PLoS One. 2024 Jul 15;19(7):e0302712. doi: 10.1371/journal.pone.0302712 (PMC11249214; doi:10.1371/journal.pone.0302712)
Supplement: Attachment — Submitted filename: LQAS methodology to quantify the WASH gap.docx [file pone.0302712.s006.docx]

Reviewer: In this study, the authors have provided insight into using the LQAS methodology to quantify the WASH gaps in Bentiu IDP camp, identify the priority areas for intervention, identify areas for resources allocation , and support advocacy efforts. This is an important study that sheds light on the plights of internally displaced camps in South Sudan, however few edits, correction and elaboration is required.

These points are expanded on in detail below.

MAJOR COMMENT

1)LINE 71-73

In 2016, inadequate WASH contributed to 60% of diarrheal deaths which could have been prevented by improving water and sanitation services [4]. Furthermore, improved hygiene, sanitation, and safe water access can also reduce neglected tropical diseases (such as schistosomiasis and Guinea worm diseases) morbidities by almost 80% [5].

A comprehensive paragraph introducing WASH (full definition before acronym )and its implication is important in the study background. This should be include a short description of the WASH initiative.

2)LINE 198

Nearly all households reported they eat from a shared plate

Is eating from a shared plate an unhygienic indicator? How was this indicator chosen? Any references ? … Have you also considered that shared eating and eating together is a cultural, community and family practice of Sudan? Please explain in detail how the above details were handled-cultural practice versus hygiene. If not kindly find a better indicator to assess hygiene.

3) LINE 176

Table 1. Socio-demographic characteristics of respondents

These table contains too few (not enough) variables to describe the study population. Give more information about the data in the samples/population….More variables --age/age group, mean of age, STD, under 5 children can be described too… new born? todlers, younger children … age in months etc. .. occupations if any? Level of education?

All these variable help in discussing the result outcome .. did education or occupation impact result..discuss?

4) Statistical Rigor

No statistically rigor in this study - only descriptives /summary statistics… do more inferential statistics … compare variables in different scenarios and discuss more on this in the discussion section.

LINE 12-105

In this study, we used the LQAS methodology to quantify the WASH gaps in Bentiu IDP camp, identify the priority areas for intervention, mobilize resources for action, and support advocacy efforts.

The study did not show or discuss how resources were mobilized for action….Rephrase to read : identify the priority areas for intervention, identify areas for resources allocation , and support advocacy efforts

MINOR COMMENTS

5)LINE 96

In the UNHCR WASH in emergency handbook and Sphere guideline, annual knowledge, attitude, and practice

Define UNHCR - in full before utilizing acronym
