# Supplementary material for: Lot quality assurance sampling survey for water, sanitation and hygiene monitoring and evidence-based advocacy in Bentiu IDP camp, South Sudan
Source: PLoS One. 2024 Jul 15;19(7):e0302712. doi: 10.1371/journal.pone.0302712 (PMC11249214; doi:10.1371/journal.pone.0302712)
Supplement: Attachment — Submitted filename: Response to Reviewers.docx [file pone.0302712.s007.docx]

Reviewer: In this study, the authors have provided insight into using the LQAS methodology to quantify the WASH gaps in Bentiu IDP camp, identify the priority areas for intervention, identify areas for resources allocation, and support advocacy efforts. This is an important study that sheds light on the plights of internally displaced camps in South Sudan, however few edits, corrections and elaboration is required.

These points are expanded on in detail below.

MAJOR COMMENT

1)LINE 71-73

In 2016, inadequate WASH contributed to 60% of diarrheal deaths, which could have been prevented by improving water and sanitation services [4]. Furthermore, improved hygiene, sanitation, and safe water access can reduce neglected tropical diseases (such as schistosomiasis and Guinea worm diseases) morbidities by almost 80% [5].

A comprehensive paragraph introducing WASH (full definition before acronym) and its implication is important in the study background. This should include a short description of the WASH initiative.

Response from the authors: Thank your review and for the feedback; we have added a paragraph introducing WASH and the implications along with WASH initiatives and the United Nations International Charter of Human Rights addressing the right to clean water and sanitation. This is added in the clean manuscript from lines 69 to 79.

2)LINE 198

Nearly all households reported they eat from a shared plate

Is eating from a shared plate an unhygienic indicator? How was this indicator chosen? Any references? … Have you also considered that shared eating and eating together is a cultural, community and family practice in Sudan? Please explain in detail how the above details were cultural practice versus hygiene. If not, kindly find a better indicator to assess hygiene.

Response from the authors: Thank you for your review and feedback. Eating together is a culture in the Nuer population (reference added in the main document. While the minimum standard for the Kitchen set according to the UNHCR ((‘Kitchen-Set_1.Pdf’, n.d.)) is one plate per person for five people, we assume to assess the availability of cooking utensils in this study and hence understand the risk of diseases transmission in the lack of strict hygiene. Hence, it was an indirect indicator of the clean and sufficient kitchen set availability, while people may still share plates regardless of how many sets they have.

3) LINE 176

Table 1. Socio-demographic characteristics of respondents

This table contains too few (not enough) variables to describe the study population. Give more information about the data in the samples/population….More variables --age/age group, mean of age, STD, under 5 children can be described too… newborn? toddlers, younger children … age in months, etc. .. occupations, if any? Level of education?

All these variable s help in discussing the resulting outcome .. did education or occupation impact the result..discuss?

Response from the Authors: Thank your review and the feedback; we have included more information on the sociodemographic characteristics relevant to this study's purpose, “quantifying the coverage of WASH services and identifying gaps for intervention”. We updated this on Table 1, starting with line 376 on the document.

We are not looking for a causal association in this study, and our focus is on the coverage of the WASH service and prioritizing intervention areas.

4) Statistical Rigor

No statistically rigor in this study - only descriptives /summary statistics… do more inferential statistics … compare variables in different scenarios and discuss more on this in the discussion section.

Response from the authors: Thank you for your review and the feedback. We have discussed using the LQAS approach and not using any inferential statistics, as our research problem is identifying the WASH gaps and prioritising intervention. We have also discussed the limitations of the LQAS methodology on its inferential statistics. The LQAS is a specialised methodology designed for monitoring, evaluation and access coverage such as this WASH survey, vaccination coverage surveys and others mentioned in the methods and limitations section of the manuscript. Our discussion is tailored to the standard target coverages from different perspectives.

LINE 12-105

In this study, we used the LQAS methodology to quantify the WASH gaps in Bentiu IDP camp, identify the priority areas for intervention, mobilize resources for action, and support advocacy efforts.

The study did not show or discuss how resources were mobilised for action….Rephrase to read: identify the priority areas for intervention, identify areas for resource allocation, and support advocacy efforts.

Response from the Authors: Thank you for your review and the feedback. We addressed the issue of resource mobilisation in line numbers 316 to 321 in the discussion, where we mentioned the replacement of the non-functioning latrines, the funding secured, and the faecal waste management. We did not include further detail on the specific organisations involved or their roles, as we don’t think it is needed to support the key message of resource mobilization.

We have the supplementary files folder where we have shown how the priority areas were selected, including maps.

MINOR COMMENTS

5)LINE 96

In the UNHCR WASH in emergency handbook and Sphere guideline, annual knowledge, attitude, and practice

Define UNHCR - in full before utilising the acronym.

Response from the Authors: thank you for your review and feedback. We have addressed the above comment in line 100 of the cleaned document.

References

‘Kitchen-Set_1.Pdf’. n.d. Accessed 22 February 2024. https://www.unhcr.org/sites/default/files/2023-06/kitchen-set_1.pdf.
